# A robust method for estimating gene expression states using Affymetrix microarray probe level data

**DOI:** 10.1186/1471-2105-11-183

**Published:** 2010-04-12

**Authors:** Megu Ohtaki, Keiko Otani, Keiko Hiyama, Naomi Kamei, Kenichi Satoh, Eiso Hiyama

**Affiliations:** 1 Department of Environmetrics and Biometrics, Research Institute for Radiation Biology and Medicine, Hiroshima University, 1-2-3 Kasumi, Minami-ku, Hiroshima, 734-8551, Japan; 2 Department of Translational Cancer Research, Research Institute for Radiation Biology and Medicine, Hiroshima University, 1-2-3 Kasumi, Minami-ku, Hiroshima, 734-8551, Japan; 3 Natural Science Center for Basic Research and Development, Hiroshima University, 1-2-3 Kasumi, Minami-ku, Hiroshima, 734-8551, Japan

## Abstract

**Background:**

Microarray technology is a high-throughput method for measuring the expression levels of thousand of genes simultaneously. The observed intensities combine a non-specific binding, which is a major disadvantage with microarray data. The Affymetrix GeneChip assigned a mismatch (MM) probe with the intention of measuring non-specific binding, but various opinions exist regarding usefulness of MM measures. It should be noted that not all observed intensities are associated with expressed genes and many of those are associated with unexpressed genes, of which measured values express mere noise due to non-specific binding, cross-hybridization, or stray signals. The implicit assumption that all genes are expressed leads to poor performance of microarray data analyses. We assume two functional states of a gene - expressed or unexpressed - and propose a robust method to estimate gene expression states using an order relationship between PM and MM measures.

**Results:**

An indicator 'probability of a gene being expressed' was obtained using the number of probe pairs within a probe set where the PM measure exceeds the MM measure. We examined the validity of the proposed indicator using Human Genome U95 data sets provided by Affymetrix. The usefulness of 'probability of a gene being expressed' is illustrated through an exploration of candidate genes involved in neuroblastoma prognosis. We identified the candidate genes for which expression states differed (un-expressed or expressed) when compared between two outcomes. The validity of this result was subsequently confirmed by quantitative RT-PCR.

**Conclusion:**

The proposed qualitative evaluation, 'probability of a gene being expressed', is a useful indicator for improving microarray data analysis. It is useful to reduce the number of false discoveries. Expression states - expressed or unexpressed - correspond to the most fundamental gene function 'On' and 'Off', which can lead to biologically meaningful results.

## Background

Microarray technology is a high-throughput method for measuring the expression levels of thousand of genes simultaneously. Recent completion of the MicroArray Quality Control (MAQC) project ensures intra-platform consistency across test sites as well as a high level of inter-platform concordance [1]. As a result, microarrays are increasingly being used in the medical and biological fields as a powerful tool for disease diagnosis, identifying biomarkers, and studying gene function. However, observed intensities combine non-specific bindings including cross-hybridization or stray signals, which is a major disadvantage of microarray data.

The Affymetrix GeneChip microarray, in which Oligonucleotides of 25 bp are used to probe genes, is designed to include measures that allow the evaluation of non-specific hybridization. Each gene will be represented by 11~20 pairs of olligonucleotides referred to as a probe set (for example, the Human Genome U95 array uses 16 probe pairs and the Human Genome U133 Plus 2.0 array uses 11 probe pairs). Each of the probe pairs in a probe set consists of a perfect match (PM) and a mismatch (MM) probe. The PM probes are designed to bind perfectly to the gene of interest and the MM probes are created by changing the middle (13^th^) base to disrupt the bulk of specific hybridization [2]. However, opinions vary regarding the usefulness of MM measures.

Background correction algorithms for the Affymetrix GeneChip microarray may be classified into two groups: those that use MM measures (e.g., dChip difference mode [3] as well as MAS5 [4] and its later, improved version *PLIER *[5]) and those that do not (dChip PM mode [6], RMA (Robust Multi-array Analysis) [7] and its modified version, PM-only GC-RMA [8]). RMA and MAS5 are representative algorithms used for background correction. With the RMA method, only PM is used to obtain a corrected intensity. MAS5 was originally provided as a default measure by Affymetrix, in which PMs are corrected by subtracting MMs, but many researchers pointed out that direct subtraction of MM from PM is unlikely to be useful [9]. The preprocessing step affects the stochastic properties of the final statistical summaries [10]. Biologists who want to analyze microarray data might be bewildered with the availability of so many preprocessing procedures with varying results [11].

Biologically, it is likely that not all observed intensities are associated with expressed genes -- that is, many of those are associated with unexpressed genes, of which measured values simply express noise due to non-specific binding, cross-hybridization, or stray signal [12]. It has been reported that only 30-40% of the genes [13] -- around 10,000-15,000 genes in total [14]-- are expressed in human cell lines *in vitro*. Identifying probe sets associated with un-expressed genes would allow the subsequent statistical analysis to be carried out with greater efficiency. For example, in an analysis aimed at finding differentially expressed genes, filtering out these probe sets prior to analysis contributed to a decreased number of false discoveries [12,15].

In previous work, we proposed a mathematical model based on the assumption that a gene has two separate functional states - 'On' means a gene is really expressed and 'Off' means a gene is un-expressed - for identifying differentially expressed genes between two cell types [16]. Furthermore, we proposed to identify 'Off' genes using an order relationship between PM and MM measures using Affymetrix GeneChip probe level data [17]. We applied the 'On/Off' model to real medical or biological data and obtained meaningful results [18-20]. In this study, we propose to quantify a gene as being expressed using a Weibull-Normal mixture distribution with two components corresponding to the separate states 'On' and 'Off'. The probability of a gene being 'On' is obtained from the posterior probability using this Weibull-Normal mixture distribution. We examine the advantage of our method over the detection call of MAS5 using the data sets of Human Genome U95 provided by Affymetrix. We implement our proposed methods of microarray analysis to explore candidate genes involved in neuroblastoma prognosis.

The symbol *X *denotes the number of pairs in a probe set satisfying *PM *>*MM *:*X *= #{*j*|*PM*_*j *_>*MM*_*j*_, *j *= 1, ⋯, *J*}, where *J *is the number of probe pairs in a probe set.

## Results

### Estimation of Weibull-Normal density function

**Figure 1:**
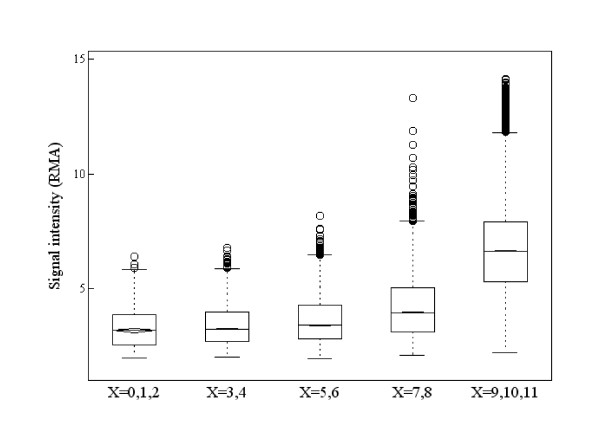
**Parallel boxplots of gene expression levels by RMA graded according to the value of *X *(number of pairs where PM>MM)**. A gene with high expression level has a large value of *X*, but large *X *does not necessary imply a high expression level; expression level of a gene with large *X *can vary from low to high.

**Figure 2:**
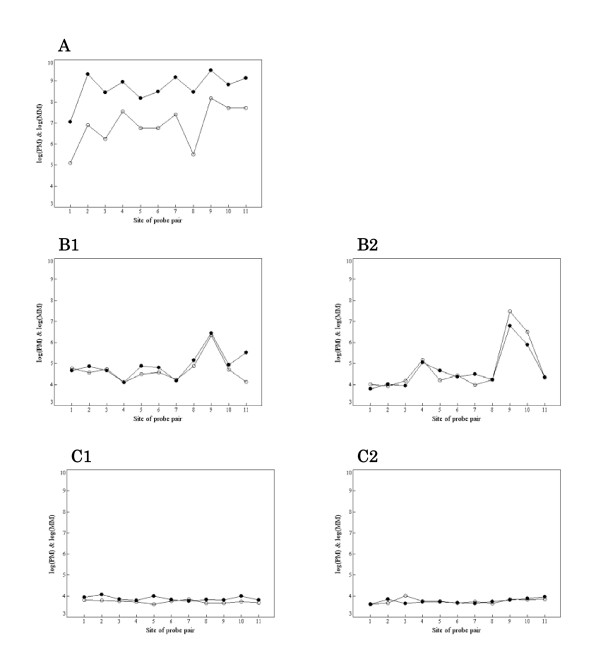
**Comparison of PM and MM measurements within a probe set**. Closed and open circles represent PM and MM measures, respectively. **(A) **High expression level. The PM value is suitably larger than MM value in every probe pair. Separating the signal of specific binding from that of non-specific binding might be possible in such a case. **(B) **Moderate expression level. The MM value is near the PM value in every probe pair; separating the signal of specific bindings from that of non-specific binding is difficult. However, the value of *X *is informative as to whether a gene is truly being expressed or not. (B1) shows 'On' (*X *= 8)) and (B2) shows 'Off' (*X *= 3). **(C) **Low expression level. It is difficult to determine whether a gene is expressed or not. (C1) shows 'On' (*X *= 10) and (C2) shows 'Off' (*X *= 6).

Figure 1 illustrates the relationship between gene expression level and the value of *X*, where the RMA summarized value was used as the measure of gene expression level (signal intensity). It shows that a gene with high expression level has larger *X *-- that is, the gene is in the 'On' state. Similarly, a gene with small *X *('Off' gene) has low expression intensity. However, not all genes with large *X *('On' genes) evidence high expression levels. Figures 2A-C show PM and MM measurements for probe sets in which summarized expression levels are high, moderate, or low, respectively. Each probe set was sampled randomly from the high, moderate, or low expression group. If gene expression level is high enough, the PM value is adequately larger than the MM value in every probe pair, and it is possible in principle to separate a signal of specific binding from one of non-specific binding (Figure 2A). In the case that the MM value is close to the PM value in every probe pair, it is presumably difficult to separate a signal of specific binding from one of non-specific binding (Figure 2B). However, the value of *X *informs as to whether a gene is truly expressed or not. Figure 2B-1 shows an 'On' gene (*X *= 8) and Figure 2B-2 shows an 'Off' gene (*X *= 3). When gene expression intensity is low, it is difficult to distinguish non-specific signal from total signal intensity (Figure 2C). Figure 2C-1 shows an 'On' gene (*X *= 10) with low intensity. Figure 2C-2 shows an 'Off' gene (*X *= 6) with low intensity. In this case, both PM and MM values represent measures of non-specific binding. Briefly, the value of *X *provides qualitative information as to whether a gene is being expressed or not and it is more informative, especially when the gene expression level is not high. We propose to quantify a gene as being expressed using a random variable *Z *derived from X and assume that *Z *follows a Weibull-Normal mixture distribution with two components corresponding to the separate states 'On' and 'Off'. The probability of a gene being 'On' is obtained from the posterior probability using this Weibull-Normal mixture distribution.

**Figure 3:**
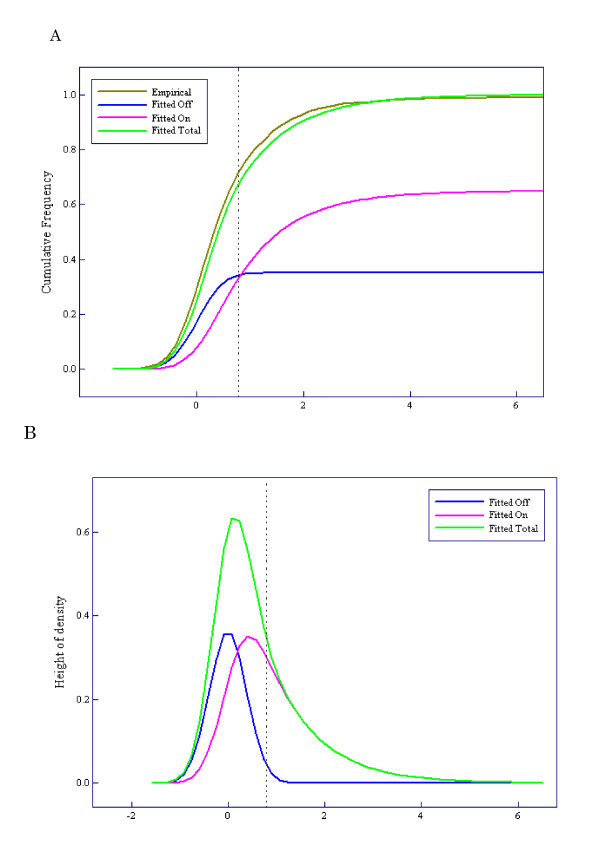
**Results of applying the Weibull-normal mixture model to the Human Genome U95 data sets**. **(A) **Comparison between the fitted Weibull-normal distribution (green line) with two components ('On' and 'Off') and the empirical distribution (brown line). The pink line shows the component of expression intensities of 'On' genes and the blue line that of 'Off' genes. **(B) **Density function corresponding to the fitted Weibull-normal distribution function (green line) and its components (pink and blue lines).

The results of applying the Weibull-Normal mixture model to the Human Genome U95 data sets are shown in Figure 3. The estimated parameter vector was  = (1.00, 1.00, 0.35, 0.15), where *μ *and *α *denote location and power parameters of the Weibull distribution, *ξ *denotes mixture rate of 'Off' genes, and *σ*^2 ^denotes the variance of the Normal distribution. Figure 3A shows a comparison of the fitted Weibull-Normal distribution with two components ('On' and 'Off') to the empirical distribution. Figure 3B shows the corresponding density function and its components. We defined the gene state as 'On' if *X *≥ 11 and 'Off' if *X *≤ 10. The vertical dotted lines in Figures 3A and 3B correspond to *X *= 11.

### Comparison between MAS5 calls and 'On/Off' calls usingspike-in genes

The MAS5 method also provides a qualitative evaluation by calling gene expression present (P), marginal (M), or absent (A) for each probe set in determining whether the measured transcript is detected or not detected. However, there is an important difference between a gene being 'Off' and a call of 'absent'. In the cases of Figure 2B-1 and 2C-1, for example, the probe sets were called 'absent' whereas their states were determined to be 'On'. To make the detection call, the MAS5 method uses a nonparametric statistical test (Wilcoxon signed rank test) under the null hypothesis that PMs and MMs have the same distribution [4]. The MAS5 method attempts to identify truly expressed genes with certainty. Exclusion probes that are called 'absent' can result in many false negatives and loss of a large amount of information, especially with genes that switch between 'On' and 'Off' with different phenotypes. On the other hand, our method seeks to correctly identify 'Off' genes using an order relationship between PM and MM measures.

**Figure 4:**
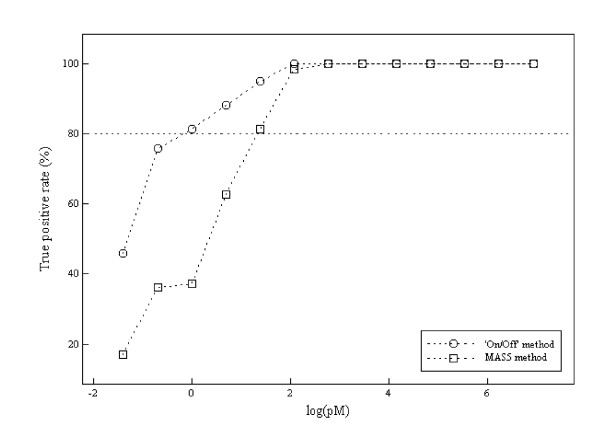
**Sensitivities of On/Off calls and MAS5 calls using the spike in genes**. The X-axis indicates log-transformed concentrations of spike-in genes; the Y-axis indicates true positive rates. Circles represent sensitivities by On/Off calls; triangles represent those by MAS5 calls.

We compared 'On/Off' calls with the MAS5 calls using spike-in genes of the Human Genome U95 data sets. The spike-in genes with 0 pM concentration were used as negative controls (N = 59). The spike-in genes with more than 0.25 pM concentration were used as positive controls (N = 767). A cutoff point dividing gene states into 'On' and 'Off' was determined as the minimum value of *X *that contains as small an 'Off' component as possible using the fitted Weibull-Normal distribution (see 'Methods'). The value *X *= 11 was obtained as the cutoff point and is shown by the vertical dotted lines in Figures 3A and 3B. Table 1 shows the distribution of number of P/M/A calls by MAS5 and number of On/Off genes for each concentration of spike-in genes. As is shown in Table 2, MAS5 calls generated many false negatives (19.0%) compared to 'On/Off' calls (8.7%). 'On/Off' calls generated 6.8% false positives, although less than 4% (the boundary p-value for defining Present calls) is desirable. The sensitivities of the 'On/Off' and MAS5 methods are shown in Figure 4. The 'On/Off' and MAS5 methods require at least 0.5 pM and 2.0 pM of spike-in genes to achieve around 80% sensitivity, respectively.

**Table 1:** Comparison between Detection Call and On/Off method using spiked-in genes.

	Detection Call			On/Off		
**True concentration (pM)**	**Present**	**Marginal**	**Absent**	***X *≥ 11**	***X *≤ 10**	**Total**

0	1	0	58	4	55	59
0.25	10	0	49	27	32	59
0.5	21	4	33	44	14	58
1.0	22	1	36	48	11	59
2	37	3	19	52	7	59
4	48	2	9	56	3	59
8	58	1	0	59	0	59
16	59	0	0	59	0	59
32	59	0	0	59	0	59
64	50	0	0	50	0	50
128	50	0	0	50	0	50
256	59	0	0	59	0	59
512	68	0	0	68	0	68
1024	69	0	0	69	0	69
Total	611	11	204	704	122	826

**Table 2:** False positive and negative rates by MAS5 and On/Off methods.

	False positive rate	False negative rate
MAS5	1/59 (1.7%)	146/767 (19.0%)
ON/Off	4/59 (6.8%)	67/767 (8.7%)

### Identification of candidate genes for predicting neuroblastoma prognosis

**Figure 5:**
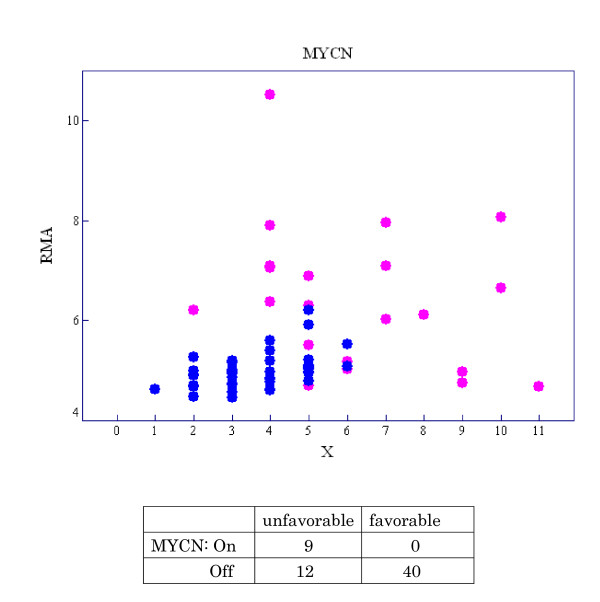
**Scatter diagram of *X *and expression intensities of *MYCN *for each neuroblastoma case**. The pink points represent cases with unfavorable outcome and the blue points those with favorable outcome. The relationship between *MYCN *state ('On/Off') and outcome (favorable/unfavorable) is listed, where the gene state 'On' is defined as *X *≥ 7 and 'Off' is defined as X ≤ 6.

Neuroblastoma is one of the most common solid tumors in childhood. Its prognosis varies remarkably, ranging from spontaneous regression to fatal progression [21]. We call these outcomes 'favorable' and 'unfavorable', respectively. It is well known that *MYCN *amplification strongly correlates with adverse outcome in neuroblastoma [22]. Nevertheless, whether *MYCN *expression is truly predictive of neuroblastoma outcome remains controversial [23]. We examined the relationship between *MYCN *expression and clinical outcomes. A scatter diagram of *X *(the number of probe pairs on an *MYCN *probe set satisfying *PM *>*MM*) versus expression intensity of *MYCN *is shown in Figure 5 for each neuroblastoma case. Pink points represent cases with unfavorable outcome and blue points represent those with favorable outcome. A cross-tabulation of state of *MYCN *being 'On/Off' and outcome (favorable/unfavorable) is also shown in Figure 5, where we define the gene state as 'On' if *X *≥ 7 and 'Off' if *X *≤ 6 according to the fit of the Weibull-Normal model. The state of *MYCN *is uniformly 'Off' in the favorable group but variable--either 'On' or 'Off'--in the unfavorable group, suggesting that *MYCN *being 'On' is sufficient for unfavorable outcome and that genes other than *MYCN *are associated with poor prognosis. We then introduce a new notation, 'OR_On' type gene, which shows the logical relationship between multiple genes and binary phenotypes.

**Figure 6:**
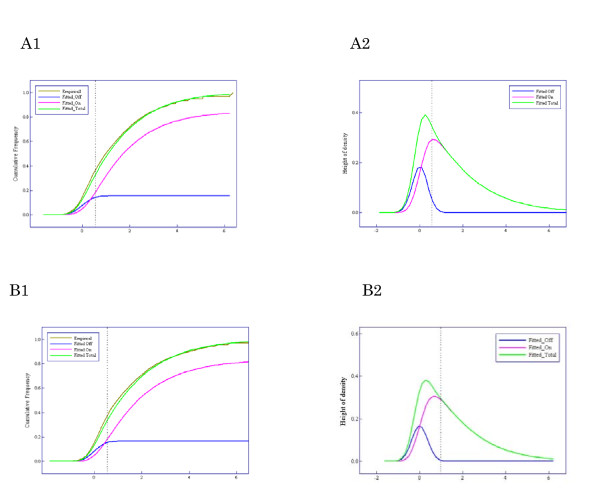
**Results of applying the Weibull-normal mixture model to the neuroblastoma data sets - A and B show the favorable and unfavorable group, respectively**. **(A1, B1)**. Comparison between the fitted Weibull-normal distribution (green line) with two components ('On' and 'Off') and the empirical distribution (brown line). The pink line shows the component of expression intensities of 'On' genes, the blue line that of 'Off' genes. **(A2, B2) **Density function corresponding to the fitted Weibull-normal distribution function (green line) and its components (pink and blue lines).

Results of applying the Weibull-Normal mixture model to 40 cases of neuroblastoma with favorable outcome and 21 cases with unfavorable outcome are shown in Figures 6A and 6B, respectively. The estimated parameter vectors for favorable and unfavorable groups were  = (1.96, 1.10, 0.17, 0.13) and (1.86, 1.16, 0.16, 0.14), respectively, where *μ *and *α *denote location and power parameters of the Weibull distribution, *ξ *denotes mixture rate of 'Off' genes, and *σ*^2 ^denotes the variance of the Normal distribution. Figures 6A-1 and 6B-1 show the fitted Weibull-Normal distribution with two components ('On' and 'Off') and the empirical distribution. Figures 6A-2 and 6B-2 show the corresponding density functions and their components. The cutoff point dividing genes into 'On' and 'Off' states, *X *= 7, is denoted by the vertical dotted lines in Figures 6A and 6B.

We set out to identify OR_On type genes involved in neuroblastoma prognosis using the estimate of the probability of a gene being 'On' or 'Off' (see 'Methods' section). A hundred genes were selected as candidates from a total of 54,109 genes. The five genes identified as candidate genes involved in neuroblastoma progression were: *MYCN *(neuroblastoma derived), *NPW *(neuropeptide W), *SLC30A3 *(solute carrier family 30, member 3), *MYCNOS *(neuroblastome derived opposite strand), and MYCN* (v-myc myelocytomatosis viral related oncogene). *MYCN *and MYCN* are the same genes detected by different probes. *MYCNOS *and *SLC30A3 *were confirmed to be correlated with the status of expression of *MYCN *in neuroblastoma [24,25]. For each of the selected genes, the probability of being 'On' in the favorable group, that in the unfavorable group, and the difference in probability of being 'On' between the unfavorable and favorable groups and its ranking, are listed in Table 3.

**Table 3:** Top five genes selected using the probability of a gene being 'On'.

	Based on the probability being 'On'			
**Gene**	**FavorablePr_On**	**UnfavorablePr_On**	**Pr_On (Unfav.) -** **Pr_On (Fav.)**	**Ranking**

*SlC30A3*	0.26	0.89	0.63	1
MYCN*	0.38	0.94	0.56	2
*MYCNOS*	0.38	0.88	0.50	3
*NPW*	0.16	0.58	0.42	4
*MYCN*	0.19	0.58	0.39	5

To assess the advantage of the 'On/Off' method, we calculated 'relative difference' statistics for the difference of average of gene expression intensities between favorable and unfavorable groups (the 'relative difference' statistic was proposed by Tusher et al [26] to stabilize the t-value). For each of the selected genes, average expression intensities obtained by the RMA methods and their standard errors in the favorable and unfavorable groups, as well as the ranking of 'relative difference' statistics in descending order, are listed in Table 4. Accordingly, the OR_On type genes are difficult to select based on the ranking of gene expression intensities. The method based on the 'On/Off' state of a gene performs better than the method based on gene expression intensity.

**Table 4:** Ranking using 'relative difference' statistics.

	RMA method		
**Gene**	**Favorable** **average (s.d.)**	**Unfavorable** **average (s.d.)**	**Ranking**

*SlC30A3*	4.93 (0.190)	5.40 (0.521)	100
MYCN**	4.59 (0.186)	5.21 (0.617)	17
*MYCNOS*	4.59 (0.152)	5.12 (0.523)	24
*NPW*	4.17 (0.106)	4.63 (0.670)	1016
*MYCN*	4.57 (0.161)	5.02 (0.508)	151

**Figure 7:**
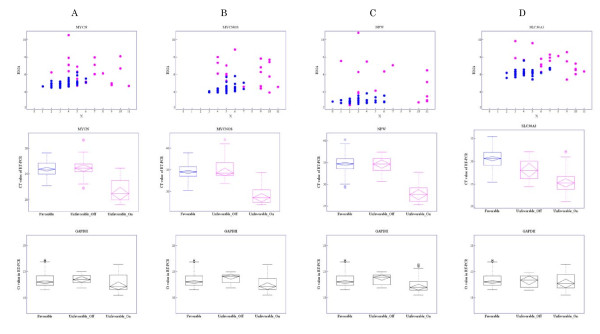
**Gene-expression features assessed by microarray and Ct values in real-time RT-PCR for four unique selected genes**. The genes selected were: A: *MYCN*, B: *MYCNOS*, C: *NPW*, and D: *SLC30A3*. The first row shows scatter diagrams of *X *versus expression intensities for each case. Pink points represent cases with unfavorable outcome and blue points cases with favorable outcome. The second row shows parallel boxplots of Ct values in real-time RT-PCR by three groups: favorable, unfavorable_On, and unfavorable_Off. The third row shows parallel boxplots of Ct values in real-time RT-PCR of *GAPDH *for the same three groups.

Real-time RT-PCR was employed to verify whether the four distinct selected genes were OR_On type genes. Gene-expression features obtained by microarray data analysis and Ct values from real-time RT-PCR for each of these genes are shown in Figures 7A-D. The displayed features are scatter diagrams of *X *and expression intensity (RMA summarized value), parallel box plots of Ct values in real-time RT-PCR for three groups -- favorable, unfavorable 'On', and unfavorable 'Off' (the latter two groups abbreviated as 'unfavorable_On' and 'unfavorable_Off ') -- and parallel box plots of Ct values from real-time RT-PCR of *GAPDH *according to the same three groups. As mentioned above, the state of a gene was defined as 'On' if *X *≥ 7 and 'Off' if *X *≤ 6. Average Ct value was significantly lower in the unfavorable_On group compared with that in the favorable and unfavorable_Off groups. This confirms that the selected genes were not expressed in the favorable and unfavorable_Off groups but were in the unfavorable_On group.

## Discussion

**Figure 8:**
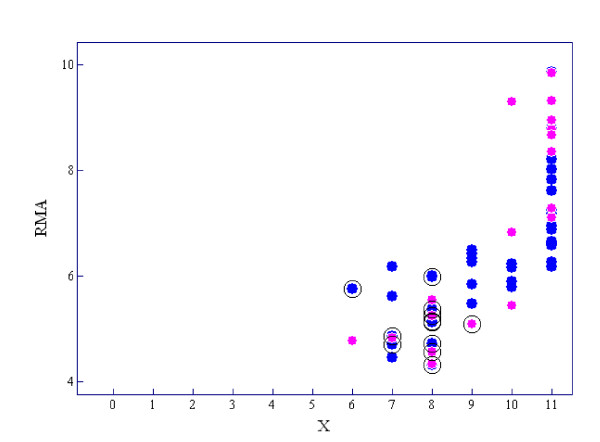
**Scatter diagram of *X *versus expression intensities of *BIRC5 *for each neuroblastoma case**. The pink points show cases with unfavorable outcome and the blue points show cases with favorable outcome. The cases called 'absent' by MAS5 are circled.

MAS5 P/M/A calls are based on a nonparametric statistical test, in which the default state of a gene is 'absent'. Therefore, it inevitably yields many false negatives which, we think, is its main disadvantage. For example, *BIRC5 *(baculoviral IAP repeat-containing 5), also called *survivin*, which is a human gene that is a member of the inhibitor of apoptosis (IAP) family, is expressed at high levels in most human tumors but is completely absent in terminally differentiated cells [27]. Figure 8 shows a diagram of *X *and expression RMA intensities of *BIRC5 *for each neuroblastoma case. *BIRK5 *was judged as 'On' in all of the 61 cases by our method but 12 cases, which are circled in Figure 8, were classified as 'absent' by MAS5. As is shown in Table 2 or Figure 8, 'On/Off' calls generated few false negatives compared to MAS5 calls. Although the poor separation of 'On' and 'Off' components of the Weibull-Normal mixture would result in false positives or negatives, we think that it is due to a limitation of microarray performance.

In this study, we only identified genes that switched 'On' and 'Off' between phenotypes using an indicator based on the probability of being 'On'. Of course, some genes exhibit differences between phenotypes in terms of their quantitative expression intensities, going from normal to abnormally increased or decreased. For example, *BIRC5 *was judged as 'On' in all cases and, in addition, 'On' with 'abnormally increased intensity' predicted poor diagnosis (unfavorable) except for one case that had been detected by mass-screening at stage I followed by radical resection. We further explored the genes whose intensity levels changed quantitatively from normal to abnormal between two different phenotypes, such as *BIRC5*, by calculating the likelihoods under the null hypothesis that gene expression intensities obey a normal distribution. As a result, more than a thousand genes were selected as candidates.

A method to create a gene expression barcode - genes expected to be expressed are coded with ones and those expected to be unexpressed are coded with zeros - was developed by Zilliox and Irizarry [28]. Furthermore, an algorithm for estimating expression states was described by McCall et al. [29]. They state that the magnitude of the unexpressed observed intensities differs among genes; accordingly the distribution of observed intensities must be estimated for each gene. Their method therefore requires a large database of observed intensities across many different tissues. We consider it natural that the magnitude of the unexpressed observed intensities differs by gene because unexpressed observed intensities include gene dependent cross-hybridization. On the other hand, our method can correct gene dependent cross-hybridizations by using MM probes. Therefore, it is unnecessary to be concerned with differences in distribution of unexpressed observed intensities among genes. Several tens of samples are enough to estimate it. Another characteristic of our method is robustness, because it is based on the order relationship between PM and MM values. Our assumptions are just (1) the expected PM value is larger than that of the MM value when a gene is expressed, and (2) the expected PM value equals that of the MM value when a gene is unexpressed. Although availability of MM probes may be in doubt [10], our approach provides good justification for the use of MMs.

## Conclusion

The qualitative evaluation 'probability of a gene being expressed' provides a useful indicator for improving the performance of microarray data analysis. When expression intensity of a gene is not high, it is difficult to determine its real intensity after removing non-specific binding. Especially in this case, 'probability of a gene being expressed' gives useful qualitative information complementing its true intensity. In regards to a practical problem in expression array analysis, genes that switch between 'On' and 'Off' with different phenotypes can be found with greater confidence. Our proposed method of estimating 'probability of a gene being expressed' is robust because it is not based on expression intensities but rather is based on the order relationship between PM and MM values.

## Methods

### Human Genome U95 data sets

The human genome U95 data sets consist of a series of 14 genes spiked-in at known concentrations (0, 0.25, 0.5, 1, 2, 4, 8, 16, 32, 64, 128, 256, 512, and 1024 pM) and arranged in a Latin square format. Each subsequent experiment rotates the spike-in concentrations by one experimental group. The data consist of 14 spiked-in genes in 14 experimental groups. Replicates within each experimental group result in a total 59 CEL files.

### Neuroblastoma samples

Total RNA was extracted from 61 neuroblastoma samples. Ages at diagnosis and stages at surgery according to the INSS (International Neuroblastoma Staging System) are shown in Additional file 1. All patients were diagnosed as having neuroblastoma between 1991 and 2005 at Hiroshima University Hospital or affiliated hospitals. Most of the patients were treated according to the Japanese neuroblastoma protocols for infants or advanced stage NB (A1, new A1, or A3) [30]. The follow-up period was more than 5 years for all patients. This research was approved by the Ethics Committee of Hiroshima University (Hiro-Rin-20). Written informed consent was obtained from parents of all patients. None of the patients had therapy prior to surgery or biopsy.

### Affymetrix microarray analysis

Microarray experiments were conducted according to standard protocols for Affymetrix Genome U133 Plus 2.0 arrays (Affymetrix, Inc., Santa Clara, CA) [31]. Briefly, using 1 μg of total RNA, cDNA and biotinated cRNA synthesis was performed using the GeneChip expression 3' amplification reagents (one-cycle cDNA synthesis, and IVT labeling) kits of Affymetrix following the manufacturer's protocols. Fragmented cRNA was applied to the hybridization and scanning of the array was performed following the manufacturer's protocols. Experimental details and all results are available at the Gene Expression Omnibus, http://www.ncbi.nlm.nih.gov/geo/ (GEO accession number GSE16237).

### Quantitative RT-PCR

In each tumor sample, cDNA was synthesized from 5 μg total RNA using a High Capacity cDNA Archive™ kit (Applied Biosystems), and then a five-hundredth aliquot of the cDNA (equivalent to 10 ng total RNA) was subjected to real-time RT-PCR using Universal Probe Library (UPL, Roche Diagnostics, Tokyo, Japan) for each target gene, or an internal control *GAPDH (glyceraldehyde-3-phosphatedehydrogenase) *TaqMan™ probe (Applied Biosystems) on an ABI PRISM™ 7900HT sequence detection system (Applied Biosystems) with 384-well plates. The relative gene expression levels were calculated as a ratio relative to *GAPDH expression level*.

### Quantification of the likelihood that a gene is 'On'

Define a random variable , the specific value of which indicates the order relationship between PM and MM: i.e.,

A set of probe level data for genes in an array *i *may be described as

We introduce a random variable *Z*^(*g*) ^defined as

to quantify the likelihood of a gene being expressed, where  is added to avoid discontinuity at  or 0 and *N *+ 1 enhances the model fit. When a gene *g *is sampled randomly, the random variable *Z *= *Z*^(*g*) ^is assumed to follow a mixture distribution with two components corresponding to the separate states 'On' and 'Off'. We assume that *Z *may be expressed as the sum of random variables *T *and *X*, where *T *expresses the likelihood of a gene being expressed when in the 'On' state and X expresses a random error having a normal density function *ϕ *with mean zero and variance *σ*^2^. We further assume that the density function of *T *is given by *f*(*t*|*μ*, *α*, *ξ*) = *ξδ *(*t*) + (1-*ξ*) *f*_*w *_(*t*|*μ*, *α*) where *f*_*w *_denotes the Weibull density function with location parameter *μ *and power parameter *α*, *δ *denotes the Dirac function, and *ξ *denotes the mixture rate of 'Off' genes. Then the density function of *Z *can be expressed as

Given a set of samples {*z*^(*g*)^| *g *= 1, ⋯, *G*}, the maximum likelihood estimate  of *θ *is obtained by maximizing . The posterior probabilities with respect to the status of gene expression can be written as

A cutoff value *z*_*c *_dividing gene states 'On' and 'Off' is determined as the lowest value of *z *satisfying Pr(*y *≥ *z *| *y *~ *ϕ *(0, *σ *^2^)) smaller than *α *(0 ≤ *α *≤ 1).

### 'AND' and 'OR' type genes

We presume the existence of 'AND' and 'OR' type genes that show a logical relationship between gene status 'On/Off' and binary phenotype. They are defined as follows:

*Definition 1*: A gene *g *is defined as 'OR_On' in the case that *g*^+ ^leads to *R*^+ ^(the gene being *on *is sufficient for unfavorable outcome), 'OR_Off' in the case that g^- ^leads to *R*^+ ^(the gene being *off *is sufficient for unfavorable outcome), 'AND_On' in the case that *R*^+ ^implies *g*^+ ^(the gene being *on *is necessary for unfavorable outcome), and 'AND_Off' in the case that *R*^+ ^implies *g*^- ^(the gene being *off *is necessary for unfavorable outcome), where the symbols *R*^+ ^and *R*- indicate the two outcome phenotypes and *g*^+ ^and *g*^- ^indicate a gene being 'On' and 'Off', respectively.

The frequency distribution of cases according to these four types of genotype/phenotype relationship can be expressed by a set of two-by-two tables (Table 5).

**Table 5:** Association between gene status and response with 'OR' and 'AND' type genes.

	OR_On		OR_Off		AND_On		AND_Off	
	R^+^	R^-^	R^+^	R^-^	R^+^	R^-^	R^+^	R^-^
g^+^	a	0	N_1_- b	N_2_	N_1_	c	0	d
g-	N_1_- a	N_2_	b	0	0	N_2_- c	N_1_	N_2_- d
Total	N_1_	N_2_	N_1_	N_2_	N_1_	N_2_	N_1_	N_2_

### Identification of 'OR_On type genes involved in neuroblastoma

We set out to identify OR_On type genes involved in neuroblastoma prognosis using the estimate of the probability of a gene being 'On' or 'Off'. OR_On type genes were identified by the following procedure.

#### Step 1

Calculate the probability of a gene being 'On' in the favorable and unfavorable groups using formula (1).

#### Step 2

A gene set *G*_1 _satisfying two conditions--(1) uniformly 'Off' in the favorable group and (2) varying 'On' and 'Off' in the unfavorable group--is defined as

#### Step 3

Arrange the values of Pr(*g *is 'Off' in the favorable group | *g ∈ G*_1_) in descending order and select the top 100 genes. Then rearrange according to

in descending order.

### Software

MAS5 and RMA expression indices were calculated using the package *affy *[32,33] provided by BioConductor [34]. Fortran was used to perform all of the analyses.

## Abbreviations

MAS5: Affymetrix Microarray Suite version 5; MM: mismatch probe; PM: perfect match probe; RMA: robust multi-array analysis.

## Authors' contributions

MO participated in the design of the study, statistical analysis, and drafting of the manuscript. KO participated in the statistical analysis and drafting of the manuscript. KH and EH were involved in conducting the microarray experiment and assisted with manuscript preparation. NK participated in conducting the microarray experiment. KS was involved in microarray analysis. All authors edited and approved the final version of the manuscript.

## Supplementary Material

Additional file 1 **Neuroblastoma cases**. The file contains the table including ages at diagnosis and stages at surgery according to the INSS (International Neuroblastoma Staging System) of 61 neuroblastoma cases. Click here for file
