# Supplementary material for: A robust method for estimating gene expression states using Affymetrix microarray probe level data
Source: BMC Bioinformatics. 2010 Apr 12;11:183. doi: 10.1186/1471-2105-11-183 (PMC2873532; doi:10.1186/1471-2105-11-183)
Supplement: Additional file 1 — Neuroblastoma cases. The file contains the table including ages at diagnosis and stages at surgery according to the INSS (International Neuroblastoma Staging System) of 61 neuroblastoma cases. [file 1471-2105-11-183-S1.DOC]

**Supplementary Table: Neuroblastoma cases**

| Case | Age at diagnosis (months) | Primary site | INSS | Outcome |
| --- | --- | --- | --- | --- |
| Favorable cases |  |  |  |  |
| 1 | 1 | retroperitoneal | 2 | NED |
| 2 | 1 | neck | 3 | NED |
| 3 | 2 | adrenal | 4S | NED |
| 4 | 3 | adrenal | 2 | NED |
| 5 | 3 | adrenal | 4S | NED |
| 6 | 3 | adrenal | 1 | NED |
| 7 | 6 | adrenal | 4S | NED |
| 8 | 6 | adrenal | 1 | NED |
| 9 | 6 | chest | 1 | NED |
| 10 | 6 | pelvis | 1 | NED |
| 11 | 6 | adrenal | 4S | NED |
| 12 | 6 | adrenal | 1 | NED |
| 13 | 6 | retroperitoneal | 1 | NED |
| 14 | 7 | retroperitoneal | 3 | NED |
| 15 | 7 | adrenal | 1 | NED |
| 16 | 7 | retroperitoneal | 1 | NED |
| 17 | 7 | adrenal | 4S | NED |
| 18 | 7 | pelvis | 1 | NED |
| 19 | 7 | adrenal | 1 | NED |
| 20 | 7 | adrenal | 3 | NED |
| 21 | 7 | adrenal | 1 | NED |
| 22 | 7 | adrenal | 2 | NED |
| 23 | 7 | adrenal | 1 | NED |
| 24 | 8 | adrenal | 1 | NED |
| 25 | 8 | adrenal | 2 | NED |
| 26 | 8 | adrenal | 1 | NED |
| 27 | 8 | adrenal | 1 | NED |
| 28 | 8 | adrenal | 1 | NED |
| 29 | 8 | adrenal | 1 | NED |
| 30 | 8 | adrenal | 4S | NED |
| 31 | 8 | adrenal | 1 | NED |
| 32 | 8 | retroperitoneal | 1 | NED |
| 33 | 9 | adrenal | 2 | NED |
| 34 | 9 | chest | 1 | NED |
| 35 | 12 | adrenal | 4 | NED |
| 36 | 16 | retroperitoneal | 1 | NED |
| 37 | 38 | adrenal | 4 | NED |
| 38 | 53 | adrenal | 2 | NED |
| 39 | 72 | retroperitoneal | 3 | NED |
| Unfavorable cases | | | | |
| 40 | 6 | adrenal | 4S | DOD |
| 41 | 9 | adrenal | 4 | DOD |
| 42 | 10 | adrenal | 4 | DOD |
| 43 | 13 | adrenal | 3 | DOD |
| 44 | 15 | adrenal | 4 | DOD |
| 45 | 15 | adrenal | 4 | DOD |
| 46 | 19 | adrenal | 4 | DOD |
| 47 | 21 | adrenal | 4 | DOD |
| 48 | 23 | adrenal | 4 | DOD |
| 49 | 23 | adrenal | 4 | DOD |
| 50 | 24 | adrenal | 4 | DOD |
| 51 | 29 | adrenal | 4 | DOD |
| 52 | 36 | adrenal | 4 | DOD |
| 53 | 38 | retroperitoneal | 4 | DOD |
| 54 | 46 | adrenal | 4 | DOD |
| 55 | 49 | adrenal | 4 | DOD |
| 56 | 59 | retroperitoneal | 2 | DOD |
| 57 | 62 | retroperitoneal | 4 | DOD |
| 58 | 64 | adrenal | 4 | DOD |
| 59 | 72 | retroperitoneal | 4 | DOD |
| 60 | 106 | adrenal | 4 | DOD |
| 61 | 117 | adrenal | 4 | DOD |

INSS: International Neuroblastoma Staging System, adrenal: adrenal gland, DOD: died of disease, NED: no evidence of disease.

The **microarray** data are available at the Gene Expression Omnibus,

<http://www.ncbi.nlm.nih.gov/geo/> (GE accession No.is GSE16237).
